# Digital elevation model and orthophotographs of Greenland based on aerial photographs from 1978–1987

**DOI:** 10.1038/sdata.2016.32

**Published:** 2016-05-10

**Authors:** Niels J. Korsgaard, Christopher Nuth, Shfaqat A. Khan, Kristian K. Kjeldsen, Anders A. Bjørk, Anders Schomacker, Kurt H. Kjær

**Affiliations:** 1 Centre for GeoGenetics, Natural History Museum, University of Copenhagen, 1350 Copenhagen, Denmark; 2 Department of Geosciences, University of Oslo, Oslo 0316, Norway; 3 DTU Space—National Space Institute, Technical University of Denmark, Department of Geodesy, 2800 Kgs. Lyngby, Denmark; 4 Department of Earth Sciences, University of Ottawa, Ottawa, Ontario K1N 6N5, Canada; 5 Department of Geology, UiT—The Arctic University of Norway, N-9037 Tromsø, Norway

**Keywords:** Cryospheric science, Geomorphology, Climate and Earth system modelling

## Abstract

Digital Elevation Models (DEMs) play a prominent role in glaciological studies for the mass balance of glaciers and ice sheets. By providing a time snapshot of glacier geometry, DEMs are crucial for most glacier evolution modelling studies, but are also important for cryospheric modelling in general. We present a historical medium-resolution DEM and orthophotographs that consistently cover the entire surroundings and margins of the Greenland Ice Sheet 1978–1987. About 3,500 aerial photographs of Greenland are combined with field surveyed geodetic ground control to produce a 25 m gridded DEM and a 2 m black-and-white digital orthophotograph. Supporting data consist of a reliability mask and a photo footprint coverage with recording dates. Through one internal and two external validation tests, this DEM shows an accuracy better than 10 m horizontally and 6 m vertically while the precision is better than 4 m. This dataset proved successful for topographical mapping and geodetic mass balance. Other uses include control and calibration of remotely sensed data such as imagery or InSAR velocity maps.

## Background & Summary

DEMs produced from aerial and satellite imagery are widely used in studies of geodetic glacier mass balance and landform mapping^1–10^. In Greenland, photogrammetry has been used on terrestrial and aerial photographs for glaciological research and topographical mapping since the early 1930s and is still in use on aerial photographs today^10–14^. Since the 1960s satellites have become an increasingly important platform for acquisition of stereoscopic imagery^15^. Gridded DEMs derived from optical imagery recorded by e.g., SPOT-5, Terra (ASTER) and WorldView satellites have been used to answer a range of outstanding research questions about the Greenlandic cryosphere^3–5^. Nevertheless, high-latitude stereo-photogrammetric DEMs, are limited by the low-visible contrast of snow and ice surfaces that reduce the ability to resolve heights from an image stereopair^6,8^. In Greenland, this limits the data acquisition to the coastal regions including the margin and outlets of the Greenland Ice Sheet (GrIS). Thus, to overcome this limitation, elevation data from other sources, such as radar altimetry, Synthetic Aperture Radar (SAR) and photoclinometry, are often combined with stereo-photogrammetry to produce a complete, but sometimes temporally inconsistent, elevation coverage of Greenland including the GrIS^7–9^.

**Figure 1:**
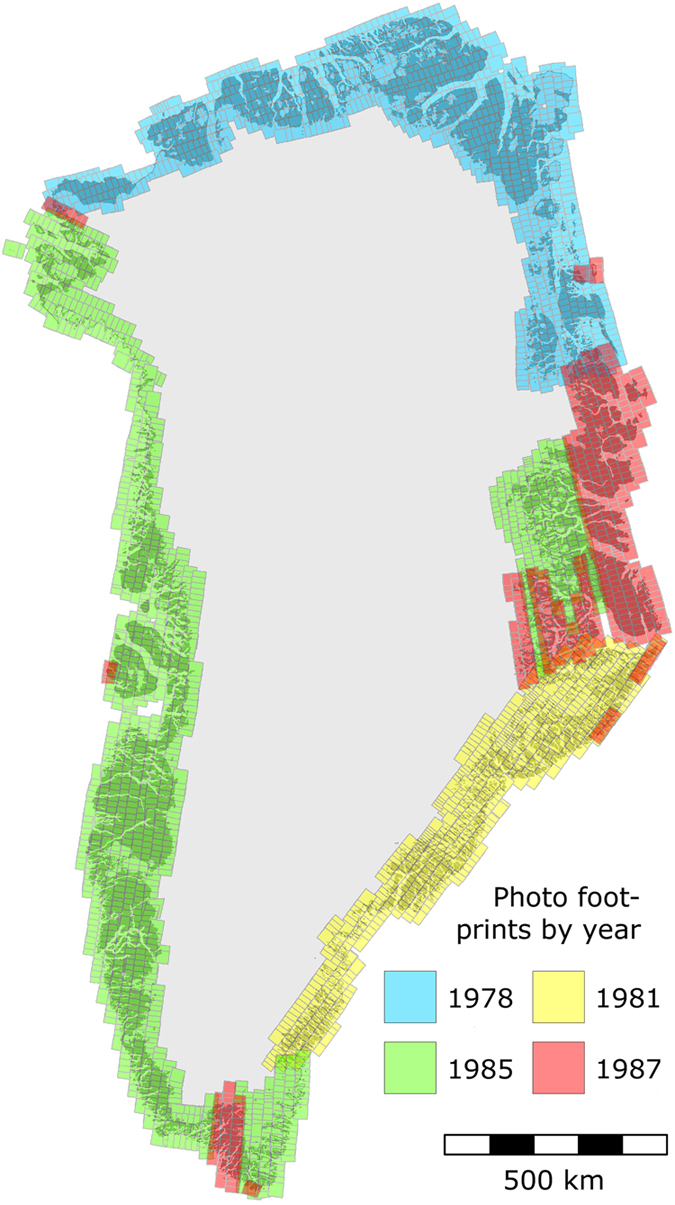
Footprints of aero-triangulated photographs by year of recording.

Here, we present a 25×25 m gridded DEM and 2 m resolution orthophotograph derived from ~3,500 vertical photographs (scale 1:150,000) acquired from 1978 to 1987 that cover all ice-free areas of Greenland and a significant part of the GrIS margin. In Fig. 1 the resulting footprints of the triangulated photos are shown by year of recording. The aerial campaigns were carried out by the Agency for Data Supply and Efficiency, SDFE (previously Geodetic Institute, then National Cadastre and Survey of Denmark, and Danish Geodata Agency), and ground control was surveyed and used for aero-triangulation of the photographs by the SDFE and the National Space Institute, Technical University of Denmark (DTU Space)^14,16–18^. A challenge for stereo-photogrammetry in remote, Arctic regions is the scarcity and quality of the ground control^6^. The photographs used for our data set have previously been used for DEMs and mapping, but geodetic control has been of inconsistent quality^7,8,12,14^. Here, we use the latest 2006 aero-triangulation with ground control provided by a subset of the GPS-based REFGR Greenlandic reference network supplemented with Doppler stations. Our technical validation comprises an assessment of the aero-triangulation, complemented by two validations using external data. We co-register DEM tiles and 50×50 km DEM blocks to ICESat laser altimetry in order to determine consistency and precision. Vertical accuracy is tested using Airborne Topographic Mapper (ATM) laser altimetry as reference data, which is also used as input to assign error to the orthophotographs.

The present DEM and orthophotograph were produced to facilitate investigations of ice sheet and glacier behavior. This is reflected in the studies where the data set has already been used, for example, the data is used to determine the geodetic mass change in northwest and northeastern Greenland, and resolved the ice sheet dynamics back to the late-1970s and mid-1980s^19–21^. Moreover, to better understand climate-related variability of glaciers in southeast Greenland, Bjørk *et al.*^11^ used orthophotographs as reference for co-registration of a wide range of imagery spanning 80 years. Khan *et al.*^22^ determined elevation change and frontal positions of Helheim and Kangerlussuaq Glaciers using heights from the DEM and orthophotographs. Lea *et al.*^23^ reconstructed Little Ice Age glacier geometry of Kangiata Nunaata Sermia in southwest Greenland. Finally, Kjeldsen *et al.*^24^ used this data set to reconstruct the geodetic mass loss of the GrIS since the Little Ice Age.

The DEM and reliability mask are made available in GeoTIFF file format, while orthophotograph and footprint coverage are made available in JPEG 2000 (jp2) and ESRI shapefile file formats, respectively.

## Methods

The materials used for our DEM and orthophotographs are the 1978–1987 aerial photos and the coordinate lists resulting from the aero-triangulation providing the geodetic control of the photographs. The super-wide-angle photographs were recorded at flying heights of 13,000 m using a WILD RC10 camera with a nominal focal length of 88 mm^16^. About 45% of the photographs were scanned on a photogrammetric film roll scanner for this work, the remainder having already been scanned on a photogrammetric film roll scanner for earlier mapping projects. Scan resolutions of 14 and 15 μm provide a nominal ground resolution of 2.1 and 2.25 m, respectively. The coordinate lists, image observations and camera calibration reports are available from the Agency for Data Supply and Efficiency^16^. The coordinate list contains the *~*21,500 Greenland Reference Frame 1996 (GR96) coordinates and heights from the aero-triangulation of the ~3,500 photographs. We use the latest aero-triangulation from 2006 for control of the aerial photographs from which we derive our DEM and orthophotograph products^18^.

### Aero-triangulation

**Figure 2:**
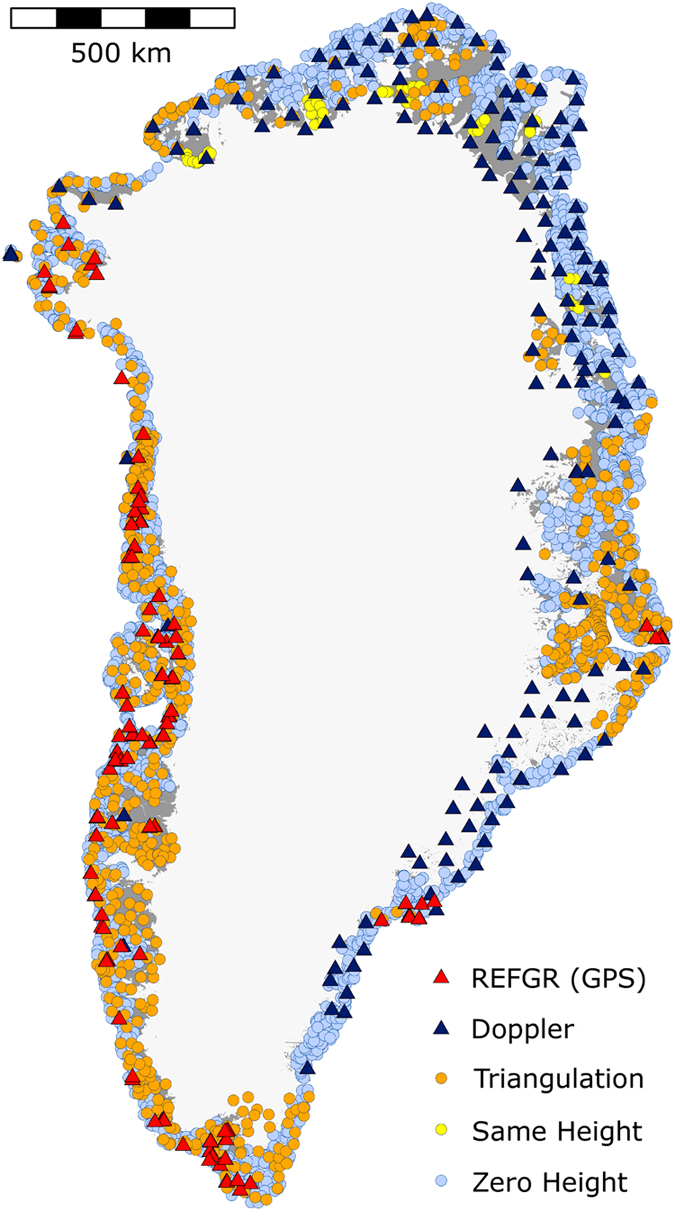
Ground control used in the aero-triangulation of the photographs. Triangulation points indicate ground control with coordinates and heights assigned by GPS or Doppler-based adjustment of the terrestrial triangulation network. Ground control outside of these networks is not connected to triangulation networks.

The ground control points for the aero-triangulation are a subset of *~*6,300 stations in the terrestrial triangulation network which had GR96 reference system coordinates assigned by GPS-based adjustment^25^. GPS stations from the Greenland Reference Network (REFGR) were used in the network adjustment allowing for a better accuracy of coordinates than previous methods permitted^14,19,25^. Coordinates in the triangulation network were recalculated, which improved accuracy to 25 cm in the denser part of the network, and 75 cm in the less dense parts^19,25^. The NAVSAT/TRANSIT Doppler stations, an elder form of satellite navigation and positioning system, were recalculated to GR96 coordinates. In general, GPS stations have been deployed in the areas covered by 1985 photos in west Greenland including Inglefield Land in the northwest, while Doppler stations have been deployed in the rest (Fig. 2). The area covered by the 1978 aerial campaign of northern Greenland has little to no terrestrial triangulation network, thus in support of the 1978 aerial photo campaign, a network of Doppler stations was deployed.

The adjustments resulted in a coordinate list, where stations had been assigned with new GR96 coordinates and heights, a subset (Fig. 2, Table 1) is used to provide geodetic ground control points for the aero-triangulation of the aerial photographs. This input list has been supplemented with special height control in the form of same height at lakes (the horizontal lake surface is used to obtain dz=0 observations) and zero height (h=0) at the coastline (Table 1).

The aero-triangulation procedure was divided into in six regional parts (1–6), in which the geodetic control was not fixed in the adjustment. Here, we rename these six parts by geography for clarity, so that part 1 is west (W), part 2 is south (S), part 3 is southeast (SE), part 4 is east (E), part 5 is northeast (NE), and part 6 is the north (N). In order to minimize tensions in the overlapping border zones, the stations in the zones were re-adjusted, while maintaining stations outside the overlap remained fixed (Table 1). The resulting mean error on coordinates and the impact of the borders is discussed in the section Internal Validation.

In the aero-triangulation, there is a good overall redundancy per station, although a few stations have low redundancy, thus the ability to detect observation errors is very good which makes the triangulation reliable.

Coordinates and heights used for the aero-triangulation were assigned *a priori* errors in order to weight the triangulation^18^. Image observations were assigned an error of 10–20 μm, while coordinates were assigned an error of 8 m. The much larger assigned error on coordinates relative to those of the survey network reflects the accuracy with which the stations (ground control) can be found in the photographs, and not the accuracy of the surveyed terrestrial network^14^. GPS heights have been assigned errors of 0.5 m, while Doppler heights range between 1 and 7 m. This is due to multiple sources of height information for these stations. The remaining triangulated heights are assigned an error of 3.5 m. Special height control, i.e., zero heights and same heights, have been assigned errors of 0.9 and 5 m, respectively.

Variance estimation is shown in Table 2, where we can assess the relationship between assigned *a priori* and *a posteriori* error estimates. Values close to one indicate agreement, however this is also dependent on the degrees of freedom in the adjustment (not shown here), which is reflected in the result. There is no weight normalization as assigned errors are based on empirical knowledge and experience, and therefore maintained. The mono errors in the regions E to N (parts 4 to 6) are smaller due to the higher *a priori* mean error of the mono observations. Weights are reduced for about 100 stations using thresholds of 3–4*σ*, to decrease the influence of large errors on the triangulation.

The ground control forming the basis of this aero-triangulation is the product of various instruments and methods. Using fixed GPS stations, DTU/SDFE recalculated the terrestrial triangulation network. Stations determined by the Doppler technique were also recalculated. Although the aero-triangulation is reliable (internally consistent) and well executed, it is also very dependent on the quality and density of the ground control.

### DEM and orthophotographs

The aero-triangulation was set up with BAE System’s digital photogrammetric application SOCET SET 5.6 for DEM and orthophotograph generation. Projects were set up in SOCET SET for each UTM zone covering Greenland (zones 19–27). Coordinates were transformed for each UTM zone and heights were transformed WGS 84 ellipsoid heights using KmsTrans2012 (ref. 26). The heights in the 25×25 m DEM grid were measured by the NGATE (Next Generation Automated Terrain Extraction) module of SOCET SET 5.6 (ref. 27). Two strategies were used for automated collection of the elevations: the standard adaptive (ngate.strategy) and a low contrast (ngate_low_sp.strategy). The latter strategy is better at sampling heights where contrast is low in the photographs. Settings were set for highest precision. The two grids were merged to one grid with priority for elevations with the highest Figure-Of-Merit (FOM), a value assigned to each height indicating how reliably it is measured. Heights and FOM values were processed to GeoTIFF files cropped along latitude and longitudes. Heights are not average heights for each 25×25 m DEM post, but rather they should be considered spot heights, i.e., the height of the 2×2 m pixel at the post^27^.

The orthophotographs are sampled using the most nadir strategy and bilinear interpolation. There is a 100 m seamline feathering along the seamlines and the photographs have been radiometrically balanced using a custom dynamic range adjustment. Geospatial Data Abstraction Library (GDAL) was used to post process the orthophotographs into jp2 format^28^.

## Data Records

**Figure 3:**
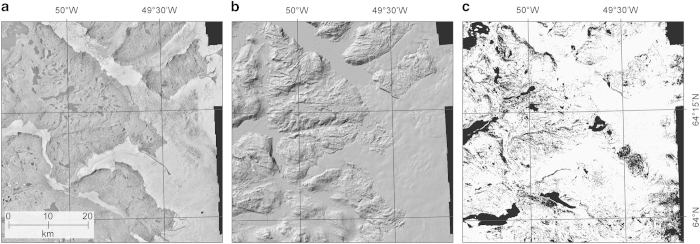
Examples of the data products from the head of Nuup Kangerlua (Godthåbsfjorden). (**a**) Orthophotograph, (**b**) hillshade DEM, and (**c**) reliability mask where white=measured heights, and black=interpolated or outside of boundary.

DEM, orthophotograph, and supporting data are archived at the National Oceanic and Atmospheric Administration (NOAA) National Centers for Environmental Information (NCEI), with access details provided in the Data Citation 1. The file formats used are GeoTIFF, jp2 (JPEG 2000), and ESRI shapefile. In addition, both raster and vector formats have an ESRI projection file. Examples of the data products are shown in Fig. 3.

### Digital elevation model

The 25×25 m gridded DEM heights are stored in 32 bit floating point GeoTIFF file format. UTM zones, predominant year of photography, and file size considerations have been used to subset the DEM into multiple files. GeoTIFF file sizes have been kept under 4 Gigabytes for user friendliness. The structure of the DEM filenames is aerodem_*year*_*utm zone*_*subset*. tif, where aerodem indicates DEM data, *year* is the predominant year of photography in the file, *utm zone* indicates native projection of the file, and *subset* indicates if DEM has been cropped due to file size considerations (Table 3).

### Reliability mask

The 25×25 m gridded reliability information is stored as 8 bit unsigned integer GeoTIFFs. The naming convention is identical to the DEM names in Table 3, but prefixed with rm. The Figure Of Merit (FOM) is a numerical value with a range of 0–100 assigned by the terrain extraction process. It may indicate one of three things for a given post measurement: It may be an error flag value, indicating that the automatic measurement was questionable; it may indicate a successful or good measurement; it may be an edit flag value, indicating the type of editing that was used (lake-filled, interpolated, etc.). As shown in Table 4 (available online only), FOM numbers greater than or equal to 40 indicate successful automatic correlation. These large FOMs are proportional to the correlation coefficient, so the larger the number, the better the measurement^29^. In summary, values in the range of 2–21 have been interpolated; values in the range 22–38 are manually edited or LIDAR points, which do not occur in the reliability mask; the value 39 is the largest value assigned to posts that did not automatically correlate; and, finally, values of 40–99 are assigned to posts which did automatically correlate with increasing quality of correlation.

### Digital orthophotograph

The 2 m resolution digital orthophotographs are stored in 8 bit JP2/JPEG 2000 (JP2ECW) file format. The naming convention of files containing the orthophotographs is identical to that of the DEM file names, with the exception that aerodem is replaced with g150 in the file name.

### Photograph ground footprint coverage

The ~3,500 photograph footprints are stored as a single ESRI polygon shapefile with reference UTM 24 / WGS 84. The dates are stored for each footprint polygon in format yyyymmdd.

## Technical Validation

In the absence of other measures of accuracy, the RMS result of a bundle block adjustment of aerial photographs is sometimes reported as an estimate of the accuracy of a photogrammetric model and its DEM product^1,21,30^. Motyka *et al.*^2^ and Khan *et al.*^20^ reported the RMS result, but subsequently used a vertical accuracy estimate based on testing of the DEM product using ATM laser altimetry as reference data. Kjær *et al.*^19^ used the same method, while Howat *et al.*^8^ applied the same method but used ICESat laser altimetry as reference to validate the GIMP DEM.

We describe and assess the precision, or model fit, of the bundle block adjustment by analyzing the spatial prediction mean error. Instead of a relatively few number of checkpoints to determine the accuracy, we use external reference data in the form of laser altimetry to validate our aerophotogrammetric DEM. We co-register entire DEM tiles and then 50×50 km DEM blocks to ICESat satellite laser altimetry to determine horizontal and vertical co-registration vectors, and RMSE. Last, we test the DEM against ATM laser altimetry to test vertical accuracy—a result we also use as input into our estimate of the horizontal error of our orthophotographs.

### Internal validation

The RMS result is a measure of model fit or precision, since the covariance matrix is an expression of the geometry of the survey network and the measurement accuracy. This assumes that the functional and stochastic models are correct: the latter assumes error to be normally distributed, observational gross errors to be down weighted or eliminated using blunder detection, and that variance estimation of groupings of observations is acceptable. There should be adequate redundancy for testing the reliability of the adjustment (Methods section).

**Figure 4:**
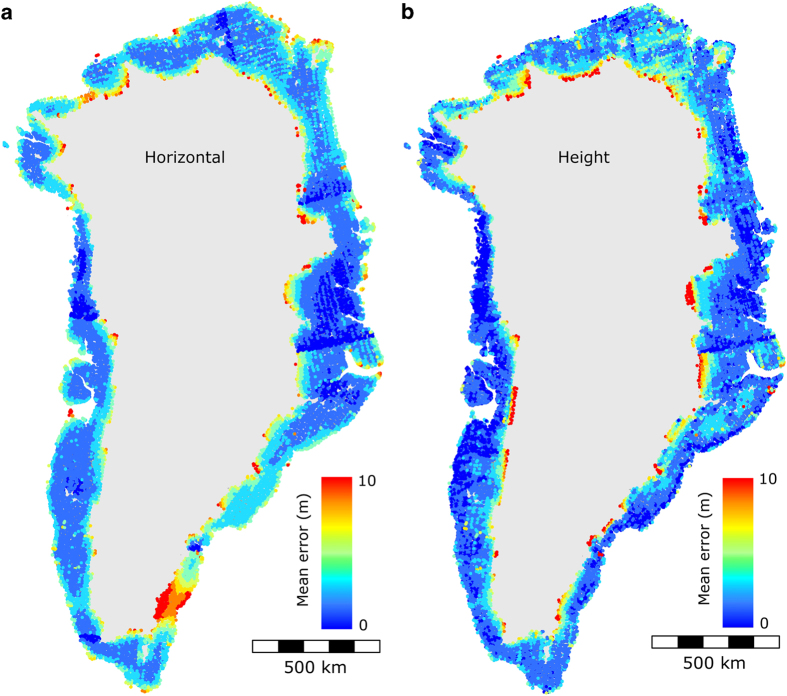
A *posteriori* mean errors from the aero-triangulation. Mean errors for both ground control points (GCP) and tie points are shown. (**a**) horizontal mean errors, (**b**) mean errors on height. Plot of the result files. Modified after Engsager *et al.*^18^.

We assess the quality and properties of the photogrammetric adjustment by examining maps of the mean horizontal and height errors (see Fig. 4). Error on coordinates and heights are found from the diagonal elements of the *a posteriori* covariance matrices resulting from the bundle block adjustment.

In Fig. 4 several patterns are apparent. First, the mean error increase with distance to ground control, i.e., the errors increase towards the interior of the ice sheet and ice caps away from the geodetic control found in the ice-free areas. The borders between the fixed and free DEM adjustment blocks are clearly seen due to the readjustment in the overlapping areas. The horizontal error map clearly shows that a section in southeast Greenland has no horizontal control, but only zero height control (Figs 2,4a).This can be recognized by the large horizontal mean errors. The color scale has been saturated above 10 m for visualization purposes. Out of the total of *~*21,500 stations, 183 (0.9% of all stations) have height errors greater than 10 m. Errors larger than 10 m have a maximum at 35 m with a mean error of 14 m. In addition, 138 stations (0.6% of all stations) have horizontal errors greater than 10 m, with a maximum of 19 m and a mean of 12 m.

### External validation: DEM co-registration to ICESat laser altimetry

This section describes the external validation of the aerophotogrammetric DEM tiles and 50×50 km blocks of the DEM around Greenland using ICESat laser altimetry, and is an expanded validation of that described in Kjeldsen *et al.*^24^. We use ICESat^31^ laser altimetry for our validation as it is one of the most consistent global elevation products available^32^. Despite the lack of full spatial coverage, each ICESat footprint of *~*60–90 m returns an elevation estimate related to the histogram peak of elevation in each footprint and are often at the decimeter precision on smooth flat surfaces^33,34^, and often better than 1–4 meters on steeper topography depending upon surface roughness^3,32,35,36^. For this particular study and validation, we use ICESat altimetry from the GLA12 Release 31 (ref. 31) product using the WGS 84 datum with ellipsoid heights in the native UTM projections that span from zones 19–27.

Our validation methodology is based upon co-registration methods described by Kääb^37^ and Nuth & Kääb^32^ that relate the 3-D co-registration vector between two elevation surfaces to terrain slope (*α*) and aspect (*ψ*). The co-registration parameters are determined by robust least squares minimizations of stable terrain elevation changes between the DEMs and ICESat (*dh*) using the following equation:

dh=a⋅cos(b−ψ)⋅tan(α)+c

Where *a* and *b* is the magnitude and direction of the horizontal co-registration vector, respectively, and *c* is the mean vertical bias between the two elevation data sources. This method uses the relationship that mis-registrations between elevation data has with terrain characteristics. It however requires a sufficient sample of elevation differences on stable terrain (i.e., non-glacier) that contains some degree of terrain slope as the flat surfaces are unsolvable and ideally a uniform distribution of terrain aspects is available.

We perform the co-registration at two different scales, the first on each aero-photogrammetric DEM tile (23 tiles) and the second on a 50×50 km grid over all the DEMs. The rectangular 50 km block size is chosen in order to have enough ICESat footprint elevations to perform the co-registration, even for those DEMs that are more than 70–80% covered by glacier ice. A 50 km block covers about the same area as the stereo coverage of a 2×4 block of photos. All slopes less than 5 degrees are removed and a curvature filter is applied to remove regions where resolution variation between the datasets may cause spurious elevation differences. The significance of the co-registration solution is strongly dependent upon the number and distribution of stable terrain elevation change points, and solutions with less than 200 points are statistically susceptible to weak solutions.

The precision and accuracy of the co-registration depends strongly on the sample of input elevation differences, the terrain characteristics, and the resolution of the input data. Previous analysis has revealed that the method is capable to co-register to *~*10% of the pixel size (resolution)^32,38^. In our study, the limiting factor is the ICESat footprint size which varies from about 60 to 90 m, and thus we predict a horizontal co-registration precision of 6–9 meters. We test this by running a minimum Monte Carlo simulation by selecting randomly a sample of elevation differences to determine the co-registration solutions for each of the grid points. These tests revealed a Root Mean Square Error (RMSE) of 3 and 1 m for the horizontal and vertical co-registration parameters, respectively.

**Figure 5:**
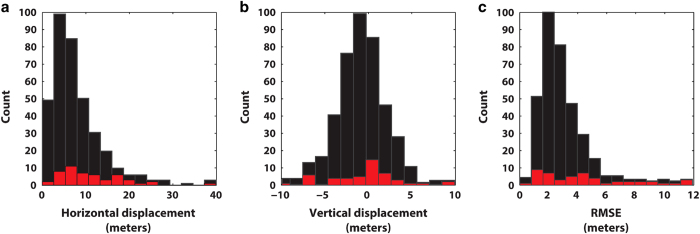
Histograms of the horizontal (**a**) and vertical (**b**) co-registration displacements for each 50 km×50 km grid cell show that the aero-photogrammetric DEM compilation is generally accurate to within 10 m horizontally and 6 m vertically with a precision greater than 4 m (1*σ* confidence level) (**c**). The red bars show the fraction of displacements determined from 200 elevation difference samples or less.

**Figure 6:**
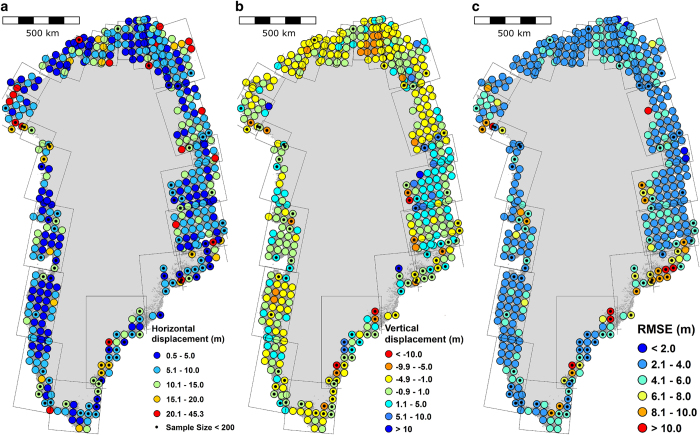
Map of the horizontal (**a**) and vertical (**b**) components of the co-registration vectors between 50 km by 50 km sections of the aerophotogrammetric DEM compilation and ICESat laser altimetry. (**c**) The RMSE of stable terrain differences after adjusting for the 3D mis-registration.

For co-registration of each entire DEM tile, all horizontal and vertical adjustments are less than 5 meters, which means that the DEMs are accurate to the precision of our method applied to ICESat data. For the denser grid, co-registration parameters are generally less than 15 meters horizontally and less than 10 m vertically. At the 1*σ* confidence level, the aerophotogrammetric DEM contains an accuracy of 10 m horizontally and 6 m vertically while the precision is better than 4 m (Fig. 5). The largest displacements between the DEMs and ICESat occur for those solutions that use rather small sample sizes (<200 elevation difference points), often those sections with the largest percent glacier cover or along the coast (Fig. 6). Although the large horizontal (and vertical) predicted mean error seen in the southeast (Fig. 4a) seems to be detected, it is questionable how confidently we can say this is due to the reliability of our method. On the southeast coast the test and reference data are a poor match, which is reflected in the small number of samples per DEM blocks. The entire southeast coast is a narrow strip of land (i.e., stable terrain), between the ice margin and the coast, and this is compounded by increasing spacing between ICESat tracks with decreasing latitude^39^.

**Figure 7:**
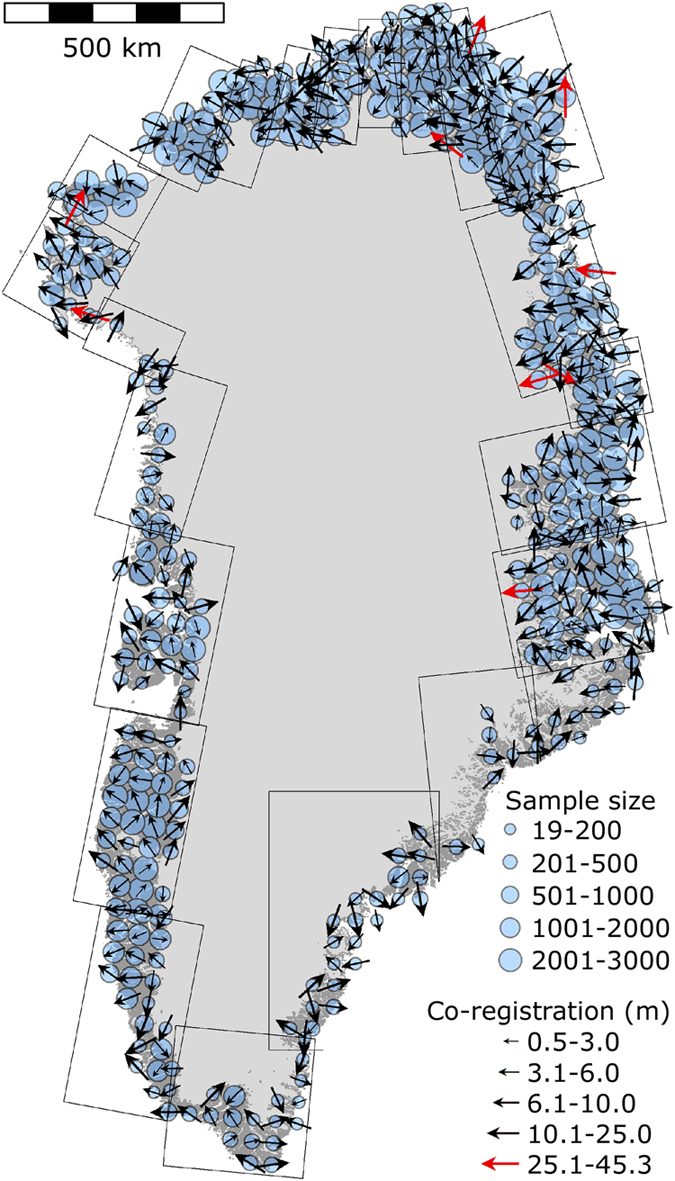
Magnitude and direction of the co-registration. There is some spatial consistency of the vertical adjustments between the aerophotogrammetric DEM and ICESat, which is likely to be related to the density of the original input ground control that is used to constrain the aerotriangulation during the adjustment of the photogrammetric model.

We plot the direction and magnitude of the co-registration vectors in Fig. 7. Vectors greater than 25 m plot along the coast or the ice margin, and are characterized by small sample sizes. Regional systematic error is apparent everywhere, but smaller and far less pronounced in the west and the south, where the ground control has the highest density. As density decrease, the ground control becomes more sensitive to the ability to identify them in the photographs and error on the coordinates, resulting in the larger magnitude and more regionalized pattern.

As an additional verification of our validation methods we use two SPOT5-HRS DEMs generated from satellite stereo images acquired in 2008 and 2014 during the IPY-SPIRIT campaign^3^. The DEMs are centered on Kangerlussuaq (1981–2008) and Daugaard Jensen Glacier (1987–2014) on the central east coast of Greenland (IDs: GES_08-013_Kangerdlugssuaq_Glacier & GES_14-012_DaugaardJensen), each scene covers more than 10000 km^2^ with about 3000 km^2^ ice-free terrain. For validation, we co-register The IPY-SPIRIT DEMs to ICESat and to the G150 DEM. While the G150 products are generally well aligned to the ICESat framework, the IPY-SPOT DEM of Daugaard Jensen Glacier has a misalignment of about a DEM pixel (40 m), which is both captured by co-registration to the G150 products as well as to ICESat. The co-registration vectors are then triangulated (vector sum) to produce a residual between the three datasets that is not larger than 4.5 meters horizontally and 0.25 meters vertically. This test strongly verifies the precision of our co-registration methods combined with the precision of the datasets used here, both aerial and satellite based.

In summary, the aerophotogrammetric DEM is consistent with the entire ICESat acquisition, at least to an accuracy of ±6 m (1*σ*), which is possible from the native resolution of the input data, constrained by the ICESat footprint size. In terms of precision of the aerophotogrammetric DEMs, our comparisons on stable terrain (that contain steep slopes) show elevation difference standard deviations for each individual DEM tile and for our 50 km co-registration grid consistently around 4 meters, or less than one fifth of the native DEM resolution. This is the maximum conservative estimate since our comparisons are based upon ICESat altimetry, and thus these estimates are the combined precision of the ICESat and aerophotogrammetric DEMs. Nonetheless, there is ample evidence that these data products are not worse than the estimates provided by this external validation, and thus these quality indicators are therefore conservative estimates which may be significantly smaller when compared with more precise validation data.

### External validation: Vertical accuracy using ATM

We test the DEM vertical accuracy with Airborne Topographic Mapper (ATM) laser altimetry from the period 1994–2014 (ref. 40). The accuracy of the reference data should be at least three times better than the data being evaluated^41^, and ATM data are therefore well suited for this purpose, as the accuracy and horizontal resolution of the ATM altimetry data are orders of magnitude better than our DEM^42^. ATM flights are flown to capture elevations on ice sheets and glaciers, thus for glaciological applications the test is often spatially close to the object of interest. The method captures the mean, random error from heights and the error induced from the horizontal displacement^43^. Kjær *et al.*^19^ found a clear relationship between slope and error in the northwestern part of the present data set, which made it possible to model vertical accuracy on ice from ATM testing on ice-free terrain. We crop the ATM data to ice-free terrain using the land coverage of GEOGREEN2 (ref. 12) with a 50 m buffer and extract elevation differences using bilinear interpolation. The resulting raw elevation differences have a Greenland-wide 1*σ*=28.7 m. We remove blunders and outliers using an 86.1 m threshold (~3*σ*) on the elevation differences. Elevation differences are then filtered using the Figure-Of-Merit greater than or equal to 40, hence we assign error to measured pixels. Finally, we also remove slopes greater than 20 degrees to assess the impact of slope-induced error. In order to assess the spatial variability of error and not bias our analysis towards areas with a higher density of flightlines, we also assign the elevation differences to a 10×10 km grid (>20 samples) shown in Table 5.

The coverage of the 1985 aerial campaign (including the 1987 photos from the west) coincide with the best ground control, while in the southeast, which was flown in 1981, the terrain is steep and rugged and more slope induced error can be expected. Therefore, we assign a vertical accuracy by predominant year of photography.

Indeed, the impact from slope-filtering on SD_ATM_ (ATM SD) is greatest on the 1981 and 1987 regions. Thus, our data suggest that this variability is best explained by a combination of horizontal displacements and the more steep and rugged terrain. In Table 5, it is also shown, that even though the ground control has similar quality in the 1978 region as for 1981 and 1987, the error can be small. The difference is due to a much less sloping terrain in the north, thereby reducing the effect of slope-induced error. Arguably, this could also be explained by the high density of samples around the Thule Base^19^; however, the statistics for the 10×10 km grid are roughly the same as for the spot heights, thus underlining that the difference is largely caused by terrain differences. We use the SD_ATM_ as input to our horizontal accuracy of the orthophotographs and consider our result as conservative, due to the ~3*σ* outlier threshold and no slope corrections. SD_ATM_ has a spread of filtered elevation differences of 1*σ*=5–10 m.

### Accuracy of orthophotographs

We calculate horizontal mean and maximum mean error using the vertical accuracy found from our ATM validation (SD_ATM_)^11,44^. The gridded DEM is TIN interpolated, but the merging of the two DEMs produced using different strategies, occasionally produces artefacts in the interpolation. We do not account for interpolation error and assign the larger maximum mean errors (6–13 m) to our orthophotographs as a conservative estimate, shown in Table 6.

### Completeness

The ability to resolve height from a stereo image-pair is dependent on high-visible contrast, continuous and unambiguous surface textures^6^. This makes it challenging to produce photogrammetric DEMs of glaciers and ice sheets in high-latitude regions due to the low-contrast surface of ice and snow on glaciers, and the shadows created by mountains and valleys^6,8^.

**Figure 8:**
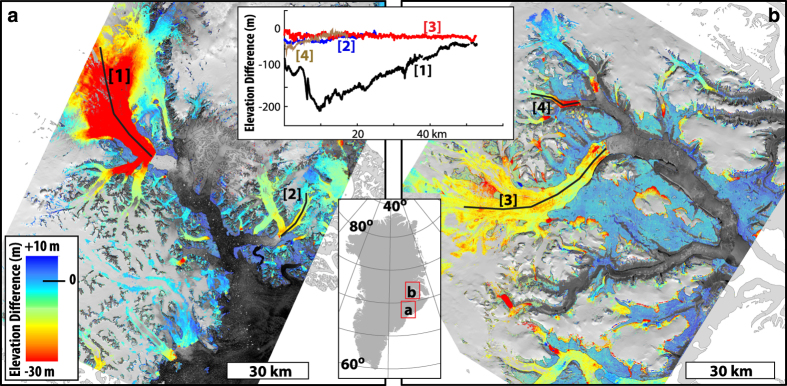
Decadal elevation change calculated using the G150 DEM and IPY-SPIRIT SPOT5-HRS^3^ DEM products. (**a**) Kangerlussuaq Glacier 1981–2008 and (**b**) Dauggaard-Jensen Glacier 1987–2014. Note the elevation difference legend has been saturated at −30 m, and elevation difference transects are plotted in the insert show actual values. Analysis of elevation change on Kangerlussuaq Glacier can be found in Khan *et al.*^22^ and Kjeldsen *et al.*^24^.

We find the completeness of two land coverages, glaciers and ice sheets (Ice/snow) and ice-free terrain/bedrock (Ice-free), by calculating the percentage of successfully resolved heights to the number of posts in each land coverage. Thus, completeness is the percentage of measurements with FOM values in the 40–99 range relative to FOM values in the 2–99 range (Table 4 (available online only)). We use GEOGREEN2 (ref. 12) map data to mask our two land coverage classes so that the coastline makes up the outer boundary and the edge of the innermost strips of aerial photographs makes up the boundary in the interior. The ICE coverage of GEOGREEN2 (ref. 12) is then used to differentiate between Ice/snow and Ice-free terrain/bedrock. The results are shown in Table 7, where it is evident that the ability to resolve heights is impacted by snow and topography. Visual inspection of the reliability masks reveal that low contrast on snow makes it difficult to resolve heights in the interior of ice sheet and glaciers, and in particular the G150 DEM products in the east (1987) and southeast (1981) are affected by this (e.g., Fig. 8). The topography is also more rugged in the east and southeast, with numerous deeply incised valleys and nunataks, which is also reflected in the results for the ice-free terrain.

### Summary of technical validation

For the internal validation we noted that the adjustment is reliable and consistent, and the spatial distribution of the mean error as expected reflects the dependence on accuracy and density of ground control and the adjustment strategy.

Our co-registration with ICESat laser altimetry shows, that at a 1*σ* confidence level the aerophotogrammetric DEM has an accuracy of 10 m horizontally and 6 m vertically, while the precision is better than 4 m. The spot height check corroborates this pattern with errors SD_ATM_ in the range of 5–10 m.

The quality of the ground control can be roughly be divided into two regions: The northwest, west and south, where coordinates and heights for a dense triangulation network have been determined using GPS based triangulation; in the southeast, east and northeast, the Doppler technique provides the coordinates and is supplemented with heights from various sources, and supported by local GPS and Doppler based triangulation networks. This pattern of deployed ground control is largely reflected in both of our external validations. Co-registration to ICESat altimetry reveals that the pattern of direction and magnitude of the co-registration parameters coincides with the spatial distribution of the ground control.

Variability in slope and ground control coincides with the reported error of the DEM obtained from comparison with ATM data. The range of the vertical accuracy is small (5–10 m), and comparable to the DEM co-registraton result. Therefore, we assign an overall accuracy of 10 m horizontally and 6 m vertically to the DEM with a precision better than 4 m, found from the DEM registration to ICESat data.

## Usage Notes

Due to the merging of two elevation grids collected with two different strategies, interpolated heights are not always consistent. For elevation change analysis, we recommend using the reliability mask as a filter and treat elevations with Figure-Of-Merit (FOM) values less than 40 in the grid as outliers.

The ESRI shapefile containing photograph footprints with recording dates is recommend for assigning dates to the observed elevations.

Orthophotographs may occasionally look ’liquid’ due to localized poor interpolation. In case of uncertainty, the reliability mask can be used to ascertain measured point density in the area of interest combined with the DEM to assess orthophotograph reliability.

### Perspectives

This circum-Greenland DEM product presents an important baseline geometric dataset for Greenland and the Greenland Ice Sheet. A number of studies have already shown the historic value of the dataset^11,19–24^, especially for estimating the recent past changes of the ice sheet and will also prove important for future studies of geometry changes and geodetic mass balance. Figure 8 shows a comparison with two DEMs acquired from the IPY-SPIRIT campaign^3^ on the central east coast of Greenland. The decadal changes are clearly visible both on the larger outlet glaciers, but also on many of the smaller glaciers and ice fields surrounding. Despite limitations of photogrammetric products on surfaces with low visual contrast that fail to achieve accurate elevations at the highest positions of the glacier, the consistency between the three data sets is remarkable, stressing the precision and accuracy of both the G150 DEM and the SPOT5 satellite DEM products but also the ability to use ICESat as a reference framework. In summary, the G150 DEM presents the first high resolution, systematic and consistent, terrain products covering the entire Greenland coastline and therefore is an important baseline dataset for future scientific discoveries. It further provides an important time stamp from the 1980s for the frontal geometry of the entire GrIS, a useful and essential product for past and future glacier volume change estimations and accurate modelling constrain.

## Additional Information

**How to cite this article:** Korsgaard, N. J. *et al.* Digital elevation model and orthophotographs of Greenland based on aerial photographs from 1978–1987. *Sci. Data* 3:160032 doi: 10.1038/sdata.2016.32 (2016).

## Supplementary Material



## Figures and Tables

**Table 1:** Number of stations, observations and redundancies

	**W**	**S**	**SE**	**E**	**NE**	**N**
Fixed stations	0	55	21	188	132	204
Free stations	4,825	4,688	4,478	3,877	4,118	4,262
Adjusted prior	0	165	2,779	706	478	644
Readjusted next	165	2,779	706	478	448	0
Mono	13,854	15,324	14,530	12,396	15,737	14,424
Coordinates	243	168	203	131	186	186
Heights	248	168	203	129	186	186
Zero or same H	775	520	499	537	588	582
Redundancies	10,402	9,970	10,207	9,720	10,096	10,145
Part 1 is the west (W), part 2 is south (S) and southeast and continues counter-clockwise. Mono is the number of image observations. Table modified from Engsager *et al.*^18^.						

**Table 2:** Variance estimation

	**W**	**S**	**SE**	**E**	**NE**	**N**
Mono	1.10	1.19	0.98	0.56	0.82	0.59
Coordinates	1.10	0.98	0.87	0.84	0.43	0.97
Heights	1.02	1.27	1.23	0.57	0.30	0.48
Determined mean error of the unit of weight on groups of observations. Table modified from Engsager *et al.*^18^.						

**Table 3:** Filename structure of the DEM GeoTIFF files

**DEM filename**	**Year of aerial photograph survey**	**UTM zone**	**Subset**
aerodem_1978_utm27_1.tif	1978	27	
aerodem_1978_utm27_2.tif	1978	27	2
aerodem_1978_utm19.tif	1978	19	
aerodem_1978_utm20.tif	1978	0	
aerodem_1978_utm21.tif	1978	21	
aerodem_1978_utm22.tif	1978	22	
aerodem_1978_utm23.tif	1978	23	
aerodem_1978_utm24.tif	1978	24	
aerodem_1978_utm25.tif	1978	5	
aerodem_1978_utm26.tif	1978	26	
aerodem_1981_utm24.tif	1981	24	
aerodem_1981_utm25.tif	1981	25	
aerodem_1981_utm26.tif	1981, 1987	26	
aerodem_1985_utm19_carey.tif	1985	19	Carey Islands
aerodem_1985_utm19.tif	1985, 1987	19	
aerodem_1985_utm20.tif	1985	20	
aerodem_1985_utm21.tif	1985	21	
aerodem_1985_utm22_1.tif	1985, 1987	22	1
aerodem_1985_utm22_2.tif	1985	22	2
aerodem_1985_utm22_3.tif	1985	22	3
aerodem_1985_utm23.tif	1985, 1987	23	
aerodem_1987_utm26_1.tif	1987, 1985	26	1
aerodem_1987_utm26_2.tif	1987, 1985	26	2
aerodem_1987_utm26_3.tif	1987	26	3
See also Fig. 4. Some tiles include a few images recorded in a different year, but the dominant year of each tiles is provided in the DEM-filename.			

**Table 4:** Definition of FOM values

**FOMVALUE**	**FOM TITLE**	**DEFINITION**
0	SMALLEST_FOM	Lowest numerical value for a FOM (set to 0).
1	OUTSIDE_BOUNDARY	Outside the extraction boundary defined as a polygon when the grid was created or defined as a rectangle. The OUTSIDE_BOUNDARY posts are not processed by any SOCET SET applications.
2	START_SUSPECT_FOM	FOMs between this value and the START_GOOD_FOMS were flagged by the correlation process and have interpolated elevations from the surrounding ‘good’ FOM points. Points with FOMs in this range (START_SUSPECT_FOM to START_GOOD_FOMS) may have good elevations since they were interpolated from the surrounding elevation data.
3	INSIDE_BOUNDARY	After the DTM is created, all FOMs will have either an INSIDE_BOUNDARY FOM or an OUTSIDE_BOUNDARY FOM. The INSIDE_BOUNDARY FOMs are assigned to points before they have gone through the correlation process or interactive edit process.
4	EXTRAPOLATED	Points which are on the edge of the DTM grid and have assigned elevations from points with good FOMs internal to the grid.
5	INTER_OPER_ BAD	INTEREST OPERATOR BAD indicates a point failed the initial correlation process and a subsequent interest operator correlation process.
6	HIGH_SLOPE	The elevation assigned to the point after correlation failed an elevation slope threshold check and has been subsequently interpolated from surrounding data.
7	TEMPORARY_FLAG	Used by software.
8	SPIKE_POST	The elevation assigned to the point after correlation failed an elevation spike or well threshold check. The spike or well was determined from the surrounding elevation data. The elevation for the point has been interpolated after it was identified as a spike or well.
9	LOW_CORRELATION_ CURVATURE	The elevation assigned to the point after correlation failed an elevation spike or well threshold check. The spike or well was determined from the surrounding elevation data. The elevation for the point has been interpolated after it was identified as a spike or well.
10	LARGE_DIFF_SIGNAL_ POWER	The signal power difference between the right and left image patches used by the correlator was lower than the threshold. The elevation for the point has been interpolated after it was identified as having a large difference in signal power.
11	EXCESSIVE_SHIFT	The maximum correlation was found on the first or last sample in the correlation image buffer. The elevation for the point has been interpolated after it was identified as having a secondary peak.
12	EDGE_OF_IMAGE	The image patch for correlation was too close to the edge of the image and could not be used by the correlator. The elevation for the point has been interpolated or extrapolated after it was identified as being on the edge of the image.
13	LOW_SIGNAL_POWER	The signal power computed for the right or left image patch in the correlator did not meet the signal power cutoff threshold. The elevation for the point has been interpolated after it was identified as having a low signal power.
14	LARGE_ELEV_CHANGE	The change in elevation during iterations within the correlator exceeds a threshold.
15	LOW_CORRELATION	The correlation coefficient computed for the point was below the threshold. The elevation for the point has been interpolated after it was identified as having a low correlation.
17	INVISIBLE	The post/point is not used to generate terrain graphics.
18	SECONDARY_ CORRELATION_PEAK	There was a secondary correlation peak identified by the correlator. The secondary peak may be almost as large as the primary correlation peak. Therefore, the point is considered as suspect. The elevation for the point has been interpolated after it was identified as having a secondary peak.
19	EXCESSIVE_V_SHIFT	The maximum correlation was found on the first or last line in the correlation image buffer. The elevation for the point has been interpolated after it was identified as having a secondary peak.
20	ELEVATED_POST	The post is identified as a none bare earth post such as on top of a tree or building. Its elevation may have been lowered to the ground by a bare earth tool.
21	START_GOOD_FOMS	Points with FOMs greater than START_GOOD_FOMS are considered good. See START_SUSPECT_FOMS for more information.
22	MANUALLY_MEASURED	The point has been edited using the Interactive Terrain Edit tools post editor or profile editor.
23–26	LAKE_FILLED, PLANE_FILLED, SMOOTHED, DWI	The point has been edited using one of the Interactive Terrain Edit area tools. Does not occur in this data set.
27	THINNED	The point has been marked as redundant by the Interactive Terrain Edit area edit tool so that it can be thinned by the DTM Export function.
28	IMPORTED_DTED	The point has been imported from DTED. Does not occur in this data set.
29	GEOMORPHIC	The point has been edited using one of the Interactive Terrain Edit geomorphic tools. Does not occur in this data set.
30	INTERPOLATED	The point has been edited using an ITE tool for clipping an area, or the point has been imported from a DTM created outside the workstation. Does not occur in this data set.
31	SEED_POINT	The point is from a see point such as a control point, or a tie point etc.
32	EDGE_POINT	This post/point is matched by edge matching.
33	CORNER_POINT	This is a corner post identified by one of the bare earth tools.
34–38	ONERETURNS,	LIDAR point/post from the first-fifth return. Does not occur in this data set.
	TWORETURNS,	
	THREERETURNS,	
	FOURRETURNS,	
	FIVERETURNS	
39	LARGEST_FOM	The largest possible numerical FOM value for a post which did not automatically correlate.
40–99		FOMs in this range indicate that the post successfully correlated. FOMs are proportional to the correlation coefficient, so the larger the number, the better the quality of the measurement.
Table is modified after the Socet Set 5.6 User Manual and reproduced with permission from BAE SYSTEMS^29^.		

**Table 5:** Elevation accuracy from spot height checks, resampling to 10×10 km grid, and mean horizontal displacement magnitude from the ICESat co-registration

**Regions by campaign year**	**1978**	**1981**	**1985**	**1987**
ATM samples	263,725	138,924	720,788	37,106
ATM samples (<3*σ*)	262,910	130,000	717,067	35,537
ATM mean (m)	1.6	−0.2	1.1	−1.5
ATM median (m)	2.0	0.2	1.0	−2.0
ATM SD (SD_ATM_) (m)	5.1	10.3	5.4	8.9
ATM mean (m) (slope<20°)	1.7	−0.4	1.1	−1.6
ATM median (m) (slope<20°)	2.1	0.1	1.1	−2.3
ATM SD (m) (slope<20°)	4.6	8.8	4.8	7.8
10×10 km mean (m) (slope<20°)	0.8	1.3	0.9	−0.8
10×10 km median (m) (slope<20°)	1.2	0.8	0.7	−1.0
10×10 km SD (m) (slope<20°)	4.1	7.8	4.5	6.6
The 1985 areas on the west coast includes the 1987 photograph recordings, and the 1987 area includes the east coast 1985 recordings (Fig. 1).				

**Table 6:** Horizontal mean and maximum mean error assigned to the orthophotographs.

**Horizontal error**	**1978**	**1981**	**1985**	**1987**
Mean error (m)	3.9	7.7	4.1	6.7
Maximum mean error (m)	6.4	13.2	6.8	11.4

**Table 7:** Completeness defined as the percentage of successful height measurements of the total possible in the photograph coverage

**Land cover**	**1978**	**1981**	**1985**	**1987**	**All**
Ice/snow, # measured heights	58,525,111	51,673,007	77,815,533	26,683,099	214,696,750
Ice/snow, # interpolated heights	104,124,608	105,690,106	89,128,630	71,333,444	370,276,788
Ice/snow, completeness (%)	36	33	47	27	37
Ice-free, # measured heights	227,871,683	80,380,392	235,502,462	112,388,069	656,142,606
Ice-free, # interpolated heights	138,718,016	127,681,459	147,065,396	102,999,959	516,464,830
Ice-free, completeness (%)	62	39	62	52	56
All, # measured heights	286,396,794	132,053,399	313,317,995	139,071,168	870,839,356
All, # interpolated heights	242,842,624	233,371,565	236,194,026	174,333,403	886,741,618
All, completeness (%)	54	36	57	44	50
The completeness on ice/snow is significantly less than for the ice-free terrain land coverage class. It is particularly evident that the combined effects of snow in the interior of the ice sheet and ice caps, and shadows in deeply incised valleys have a large impact on overall completeness in the southeast (1981) and east (1987).					
